# Variety and phosphate fertilizer dose effect on nutrient composition, in vitro digestibility and feeding value of cowpea haulm

**DOI:** 10.1186/s40781-016-0103-7

**Published:** 2016-06-01

**Authors:** Terry Ansah, Henry Ayindoh Algma, Herbert Kwabla Dei

**Affiliations:** University for Development studies, Faculty of Agriculture, Department of Animal Science, Tamale, Ghana

**Keywords:** Voluntary intake, Cowpea haulm, In vitro gas production, Djallonke, Phosphate fertilizer

## Abstract

**Background:**

Cowpea (*Vigna unguiculata* [L.]) is a legume cultivated throughout most tropical countries and is valued as food and feed for human and livestock respectively. The search for an improved cowpea variety has been on-going with the aim of improving traits such as grain yield, drought and pest resistance. But no information exist on the feeding value of these improved varieties. Phosphate (P) fertilizer application is recommended to augment grain yield in grain legumes but data on the effect of P fertilizer on haulm quality is limited.

**Results:**

Two separate experiments were conducted to determine the effect of P fertilizer dose on the nutritive value of haulms from different cowpea varieties (V). In experiment 1, effect of three P doses (30, 60 and 90 kg P_2_O_5_/ha) on in vitro gas production (IVGP) characteristics, concentrations of digestible organic matter (DOM), crude protein (CP), acid detergent fiber (ADF) and neutral detergent fiber (NDF) of haulms from five cowpea varieties (Zaayura-SARC 4-75, Songotra-IT97K-499-35, Hewale-IT93K-192-4, IT99K 573-1-1 and Asomdwe-IT94K-410-2) were investigated using the 3 (P) x 5 (V) factorial treatment arrangements in a completely randomized design. In experiment 2, the effects of two P doses (30 and 90 kg P_2_O_5_/ha) and two varieties (Zaayura-SARC 4-75 and Hewale-IT93K-192-4) on the voluntary feed intake, live weight, haematology and carcass characteristics of Djallonke lambs were also assessed using a 2(P) x 2(V) factorial treatment arrangement. The V x P interaction significantly affected CP, NDF and ADF with CP concentration increasing with increase in P doses in Zaayura-SARC 4-75 and Asomdwe-IT94K-410-2. Whilst an increase (*P <* 0.05) in NDF was observed in Songotra-IT97K-499-35and Asomdwe-IT94K-410-2 as P doses increased, the other V only increased from P dose 30 to 60 kg/ha and declined at P dose 90 kg/ha. The ADF decreased (*P* < 0.05) with increase in P dose for all V with the exception of Songotra-IT97K-499-35. There was a significant V effect on DOM with the highest reported in Zaayura-SARC 4-75 (43.44 %). Daily DM intake, carcass length and blood urea nitrogen of the lambs were significantly affected by the V x P interaction. There was a significant V effect on globulin and P effect on live weight at slaughter, dressed weight, chuck, leg, loin, rib and flank and liver and lungs.

**Conclusion:**

It can be concluded that nutrient concentrations of cowpea haulms were positively influenced by different P dose and varieties with favorable effects on growth, haematology and carcass composition of lambs.

Varieties Zaayura-SARC 4-75 and Hewale-IT93K-192-4 at P dose at 90 kg/ha are recommended to enhance growth performance and carcass yield of Djallonke lambs.

## Background

Cowpea (*Vigna unguiculata* [L.]) is a legume cultivated throughout most tropical countries and is valued as food and feed for human and livestock respectively. It is drought tolerant, can be grown on relatively poor soils, and fixes nitrogen, thereby improving soil fertility [1]. Mixed farming is a common farming system in most tropical countries. Livestock in mixed farming systems are usually fed with residue from crop fields to sustain growth during periods of feed shortage.

Leguminous haulm like cowpea haulms are a major source of dietary protein supplement to ruminants fed basal diets from cereal residues or grazing natural pastures during the dry season in smallholder livestock systems [2]. Calves fed a basal diet of teff straw and supplemented with cowpea haulm were found to have an increased supply of microbial nitrogen [3]. In another study, where ewes were fed maize stover supplemented with cowpea haulm, DM intake, nutrient digestibility and rumen ammonia concentration were found to have improved [4].

Several dual purpose cowpea varieties have been bred with the aim of improving grain yield, pest resistance, and drought tolerance [5]. Investigating the nutritive value of the haulms from these new varieties is justified considering the important role they play in the livestock industry.

Application of phosphate (P) fertilizer has been shown to increase grain yields in cowpea [6] but limited information exists on its effect on the feed value of haulms from different varieties in the tropics. Phosphate fertilization was found to affect crude protein, neutral detergent fiber and acid detergent fiber of sainfoin (*Onobrychis sativa* [L]) [7].

The in vitro gas production technique has over the years been proven to be the most reliable, simple and efficient way of evaluating feedstuff for their potential in the animal industry [8–10]. Rumen fluid for in vitro gas studies is often obtained from fistulated animals. However, in most African research institutions, these animals are normally not available mainly due to the cost of management and welfare. Some researchers have successfully used rumen fluid from slaughtered animals as substitute for the fistulated ones [11, 12]. The results were highly correlated with Tilley and Terry In vitro organic matter digestibility (IVOMD) (*R*^*2*^ = 0.73) and ME (*R*^*2*^ = 0.92).

The objectives of this study were therefore to:

1. Determine the effect of three P doses on the CP, NDF, ADF and gas production characteristics of haulms of five varieties.
2. Determine the effect of two P doses on dry matter intake, daily weight gain, carcass characteristics and blood biochemistry of Djallonke rams supplemented with haulms of two cowpea varieties.

## Methods

### Study area

The cowpea varieties were cultivated in Tingoli and Tibali in the Tolon and Savelugu Districts respectively, and are located in the Northern Region of Ghana. According to the Meteorological Services Departmenyt of Ghana, the Tolon District is characterised by a unimodal rainfall pattern with an average plant growth period of 183 days. The average rainfall per annum is 1057 mm. The Savelugu District has an average rainfall of 1067 mm with an average plant growing period of 181 days.

The varieties (Zaayura-SARC 4-75, Songotra-IT97K-499-35, Hewale-IT93K-192-4, IT99K 573-1-1 and Asomdwe-IT94K-410-2) were cultivated on an area of 1200 m^2^ with each variety occupying a total area of 300 m^2^. The intra and inter row spacing was 75 cm × 20 cm. The phosphate fertilizer (P_2_O_5)_ was applied 14 days after planting. They were harvested at flowering stage and air dried for 3 days after which they were packed and stored under shade for the trial.

The chemical analysis, in vitro gas production study and lamb growth trial were conducted at the Faculty of Agriculture, University for Development Studies (UDS), Nyankpala Campus, Tamale.

### Experiment 1

The 5*3 factorial in completely randomized design was used for the nutrient composition study. The factors were the five varieties of cowpea (Zaayura-SARC 4-75, Songotra-IT97K-499-35, Hewale-IT93K-192-4, IT99K 573-1-1 and Asomdwe-IT94K-410-2) and the three phosphate fertilizer doses (30 kg/ha, 60 kg/ha and 90 kg/ha) and were replicated 4 times.

Air dried samples of the haulm were taken and milled through 1 mm sieve screen and oven dried over night at 60 °C for the chemical analysis.

Total Nitrogen (N) was determined using the micro Kjeldahl after which the CP was calculated by multiplying the N content by 6.25 [13]. The NDF was determined exclusive of residual ash with sodium sulfite and α- amylase whilst ADF was determined exclusive of residual ash. The procedure of Van Soest et al. [14] was followed and was run on the Ankom^200^ fiber analyzer.

The 5*3 factorial in randomized complete block was used for the in vitro gas production experiment with each treatment replicated 2 times in each period. The factors were the five cowpea varieties and the three phosphate fertilizer doses. There were 4 periods in all and each period lasted for 48 h. Period was used as block.

Approximately 200 mg of the milled and oven dried samples were weighed into 50 ml test tube for incubation. Buffer was prepared following the procedure of McDougall [15] and placed in a water bath with continuous supply of carbon dioxide (CO_2_) until the rumen fluid was ready. Rumen fluid was collected from the rumen of 3 separate cattle after slaughter into pre-warmed vacuum at the Tamale abattoir. The rumen fluid was strained through a four layer cheese cloth and mixed with the buffer in a ratio of 1:4 with continuous supply of CO_2_ to get the buffered rumen fluid. Approximately 30 ml of the buffered rumen fluid was dispensed into each test tube containing the samples using a 50 ml syringe and placed in water bath at 39 °C.

The gas production was measured using a digital manometer at 3, 6, 12, 24 and 48 h. The contents of the test tubes were agitated each time the gas was measured.

The gas measured was fitted to the exponential model of Ørskov and McDonald [16] (Y = b (1 – e-^ct^) to derive the degradation characteristics (b and c) where,

- Y = gas volume at time t (ml)
- b = asymptotic gas production (ml)
- t = time (h)
- c = fractional rate of gas production (ml/h)

Digestible organic matter (DOM) was calculated using the equation of Menke and Steingass [9]. DOM (%) = 16.49 + 0.9042 GP + 0.0492 CP + 0.0387ash

Where,

- GP = gas production (ml/200 mg DM at 24 h)
- CP = crude protein (g/kg DM)

### Experiment 2

#### Lamb growth study

Two of the cowpea varieties (Zaayura-SARC 4-75 and Hewale-IT93K-192-4) and two phosphate doses (30 kg/ha and 90 kg/ha) were selected for the lamb growth trial. These were selected based on their slightly superior in vitro digestibility.

The 2*2 factorial in a completely randomized design was used for the lamb growth study. The factors were the two varieties of cowpea (Zaayura-SARC 4-75 and Hewale-IT93K-192-4) and two phosphate fertilizer doses (30 kg/ha and 90 kg/ha). There were 5 replicates (lambs) per treatments.

A total of 20 intact male Djallonke lambs (8-12 months old) with an initial average live body weight of 15.8 ± 2.3 kg were purchased from different livestock markets in the Northern Region. The animals were housed individually in wooden cages with concrete floors. The cages were fitted with wooden feeding troughs and plastic watering bowls. The animals were fed with the haulms *ad libitum* at 07:00 h and released for grazing on natural pastures at 10:00 h. Water was also given *ad libitum* and was replaced at the same time of feeding. The animals were allowed 2 weeks period of adjustment to feed and cage.

#### Supplementary feed intake

Each week, 1 kg of each treatment was weighed into jute sacks from which the animals were fed daily. Samples of the haulms in the sack were taken daily and stored in a freezer until the experiment was over. After the experiment, the sampled feed was bulked for each treatment and subsamples taken for oven drying at 60 °C for 48 h.

The daily feed refusals in the trough were collected and weighed. After the entire experiment, the total feed refusal was subtracted from the total feed offered and expressed on DM basis to get the supplementary feed intake.

#### Weekly live weight

The weekly live weight of the animals were measured using a hanging scale (Camry hanging scale, ISO9001:2008, China). From the weekly live weight, the daily live weight gain was estimated by subtracting the initial weight from the final weight for each animal and dividing by the total number of days (56 days).

#### Blood collection and processing

Blood samples were taken at the end of the experiment for the haematology and blood metabolites. The blood samples were taken at about 07:00 h. Approximately 10 ml of blood was taken with a syringe from the jugular vein and transferred into two sets of test tubes, one without anti-coagulant for the blood metabolite and the other with anti-coagulant (Ethylene di amine tetra acetic acid (EDTA)/ fluoride) for the haematological parameters. The blood sample without anti-coagulant was centrifuged and the serum separated. The serum was then transferred into clean test tubes and stored at 4 °C for the albumin, total protein, glucose and blood urea nitrogen (BUN) analysis. Globulin was calculated from total protein and albumin. The blood sample with anti-coagulant was used for the analysis of haemoglobin (Hb), white blood cells (WBC) and parked cell volume (PCV).

#### Carcass characteristics

Three animals were selected from each treatment group for the carcass analysis at the Meat Unit of the University for Development Studies, Tamale. Feed was withheld from the animals 24 h before slaughter and their weights taken to represent the slaughter weight. All animals were slaughtered and cut into parts on the same day. Immediately after slaughter, the skin, head, feet and testes (external organs) were removed and weighed. The dressed weight, dressing percentage and carcass length were taken and together with the slaughter weight were referred to as carcass parameters. The carcasses were eviscerated and the internal organs were removed and separately weighed. The internal organs (non-carcass characteristics) measured included, empty digestive tracts, liver, kidney, lungs, heart and spleen. Hot Carcass weight was taken after all the internal and external organs have been removed. The carcass was then cut into neck, shoulder, rib and flank, back, chuck, leg and loin.

#### Statistical analysis

The data from the chemical composition, in vitro gas and lamb growth were analysed as a 2 way ANOVA in Genstat 12th edition. In the in vitro *gas* study, period was used as block whereas in the lamb growth study, initial weight was used as a covariate in the analysis of the live weight.

## Results

### Experiment 1

The effect of V, P dose and their interactions on CP, NDF and ADF are presented in Table 1. The V x P interaction affected (*P <* 0.05) the CP, NDF and ADF. The highest (*P <* 0.05) CP was recorded in Zaayura-SARC 4-75 at a P dose of 60 kg/ha with the least recorded in IT99K 573-1-1 at a P dose of 60 and 90 kg/ha. The CP at a P dose of 30 kg/ha was consistently higher (*P* < 0.05) than the P dose of 90 kg/ha for all the V. The highest NDF was obtained in Songotra-IT97K-499-35 at a P dose of 90 kg/ha with the least reported in Hewale-IT93K-192-4 at 90 kg/ha. The ADF was in the range of 282 g/kg DM and 352 g/kg DM for Zaayura-SARC 4-75 at 60 and Songotra-IT97K-499-35 at 90 kg/ha respectively.

**Table 1:** Cowpea variety and phosphate fertilizer dose (kg P_2_O_5_/ha) effects on crude protein (CP), neutral detergent fibre (NDF), acid detergent fibre (ADF) concentrations (g/kg) and *In vitro* gas production characteristics of haulms (g/kg)

Item	Za			So			He			IT			AS			SED	*P* -value		
	30	60	90	30	60	90	30	60	90	30	60	90	30	60	90		V	P rate	V x P rate
CP	153	166	149	138	129	133	146	140	143	145	126	126	145	150	145	6.3	0.001	0.119	0.044
NDF	478	481	462	489	545	554	503	508	460	491	512	507	480	518	533	20.3	0.001	0.032	0.020
ADF	293	282	290	320	348	352	350	341	302	317	306	303	308	310	322	15.4	0.001	0.031	0.038
DOM	436	442	425	416	430	416	419	438	420	443	433	426	418	404	411	5.0	0.001	0.087	0.290
B	192	174	191	188	193	172	188	195	193	191	194	189	184	173	177	6.0	0.217	0.405	0.310
C	0.07	0.07	0.06	0.06	0.07	0.07	0.06	0.07	0.07	0.07	0.06	0.06	0.06	0.06	0.07	1.0	0.855	0.410	0.740
IVGP 48 h (ml/g DM)	18.4	16.8	18.3	16.4	18.8	16.8	16.6	18.8	17.7	17.3	15.6	17.1	17.4	15.6	17.2	7.0	0.241	0.849	0.209

*CP* Crude protein, *NDF* Neutral detergent fiber, *ADF* Acid detergent fiber, *V* Variety, *P* Phosphate fertilizer dose, *Za* Zaayura-SARC 4-75, *So* Songotra-IT97K-499-35, *He* Hewale-IT93K-192-4, *IT* IT99K 573-1-1, *As* Asomdwe-IT94K-410-2), *DOM* Digestible organic matter, *b* Potential degradability, *c* Rate of degradation, *IVGP* In vitro gas production, *SED* standard error of difference, *P* probability

The V x P interaction was not significant for digestible organic matter (DOM), in vitro gas production (IVGP) and rate of gas production (c). There was a significant V effect on mean DOM. The DOM ranged from 404.0 g/kg DM to 443.0 g/kg DM for varieties Asomdwe-IT94K-410-2 and Zaayura-SARC 4-75 respectively. The potentially degradable dry matter (g/kg DM), rate of degradation (c) and in vitro gas production at 48 h did not differ by V or by P (Table 1).

### Experiment 2

The results of the effect of V, P and interactions on the lamb growth parameters are shown in Table 2. The V x P interaction affected (*P <* 0.05) the daily dry matter intake (DMI). The highest DMI was reported in lambs that fed on Hewale-IT93K-192-4 at a P dose of 30 kg/ha and Zaayura-SARC 4-75 at a P dose of 90 kg/ha. The other growth parameters were not affected by the treatment interaction or the main effect.

**Table 2:** Effect of haulms of cowpea variety and phosphate fertilizer dose on growth and carcass characteristics of Djallonke lambs

	Zaayura		Hewale		SED	*P*_*-value*_		
Intake and weight gain	30	90	30	90		V	P rate	V x P rate
Daily DM intake (g/h/d)	125.5	143.9	145.1	127.7	6.9	0.621	0.730	0.003
ADWG (g)	35.5	45.1	44.6	49.7	7.6	0.185	0.213	0.694
Slaughter Weight (kg)	14.2	19.8	16.1	18.5	1.3	0.758	0.003	0.131
Dressed Weight (kg)	5.6	7.7	6.2	7.1	0.5	0.976	0.001	0.097
Dressing %	39.3	38.9	38.5	38.4	1.8	0.592	0.841	0.915
Carcass Length (m)	0.4	0.6	0.5	0.5	0.0	0.132	0.003	0.039

*ADWG* Average daily weight gain, *ADW* Average daily weight, *DMI* Dry matter intake, *BW* Body weight, *V* Variety, *P* Phosphate fertilizer dose, *DW* Dressed weight, *LW* Live weight, *SED* standard error of difference, *P* probability, *Zaayura* Zaayura-SARC 4-75, *Hewale* Hewale-IT93K-192-4

There was a V x P interaction on carcass length with the animals on V at 90 kg/ha having the longest (*P <* 0.05) carcass length (Table 3). There was a significant P effect on the slaughter weight and dressed weight with the higher P dose having a greater (*P <* 0.05) influence.

**Table 3:** Effect of haulms of cowpea variety and phosphate fertilizer dose on primal cuts

Primal cuts (kg)	Zaayura		Hewale		SED	*P- value*		
	30	90	30	90		V	P rate	V x P rate
Back	0.3	0.4	0.4	0.4	0.1	0.612	0.208	0.111
Chuck	0.4	0.5	0.4	0.6	0.1	0.439	0.003	0.753
Leg	0.8	1.2	0.9	1.1	0.1	0.955	0.003	0.051
Loin	0.3	0.4	0.4	0.4	0.1	0.424	0.017	0.310
Neck	0.5	0.7	0.5	0.5	0.1	0.147	0.112	0.097
Rib and Flank	0.4	0.6	0.6	0.6	0.1	0.089	0.008	0.197
Shoulder	0.5	0.7	0.6	0.7	0.1	0.617	0.004	0.157

*V* Variety, *P* Phosphorus fertilizer, *DW* Dressed weight, *LW* Live weight, *SED* Standard Error of Difference, *P* probability, Means with the same letters within rows are not significantly different (*P* > 0.05), *Zaayura* Zaayura-SARC 4-75, *Hewale* Hewale-IT93K-192-4

The V x P interaction was not significant on the primal cuts. However, there was a significant main effect of P dose on primal cuts. The lambs that fed on P dose of 90 kg/ha had higher (*P <* 0.05) back, chuck, leg, loin, shoulder and rib and flank than those on the P dose of 30 kg/ha.

There was a significant P dose effect on the liver and lungs among the internal organs with animals on the 90 kg /ha P dose having the highest (*P* < 0.05) (Table 4).

**Table 4:** Effect of haulms of cowpea variety and phosphate fertilizer dose on internal and external organs of Djallonke lambs

Internal organs (kg)	Zaayura		Hewale		SED	*P-value*		
	30	90	30	90		V	P rate	V x P rate
Liver	0.3	0.4	0.3	0.4	0.1	0.924	0.029	0.403
Lungs	0.2	0.3	0.3	0.3	0.03	0.048	0.048	0.134
Empty digestive tract	0.6	0.7	0.7	0.7	0.1	0.252	0.130	0.395
Heart	0.1	0.1	0.3	0.1	0.2	0.349	0.349	0.324
Spleen	0.04	0.05	0.05	0.04	0.009	0.803	0.803	0.461
Kidney	0.07	0.07	0.07	0.08	0.008	0.580	0.122	0.580
External organs (kg)								
Testis	0.3	0.3	0.3	0.4	0.05	0.523	0.416	0.782
Skin	1.0	1.4	1.3	1.3	0.11	0.969	0.014	0.373
Head	1.2	1.7	1.3	1.5	0.01	0.329	0.001	0.028

*V* Variety, *P* Phosphorus fertilizer, *SED* Standard Error of Difference, *P* probability, Means with the same letters are not significantly different (*P* > 0.05), *Zaayura* Zaayura-SARC 4-75, *Hewale* Hewale-IT93K-192-4

The haematology and blood biochemistry were not affected by the V x P rate interaction and main effects, except for the globulin and blood urea nitrogen (BUN). Animals supplemented with Hewale-IT93K-192-4 at a P dose of 90 kg/ha had higher (*P <* 0.05) BUN compared with the other treatments (Table 5). Similarly, animals on Hewale-IT93K-192-4 had higher (*P <* 0.05) globulin concentration compared to the other varieties.

**Table 5:** Effect of haulms of cowpea variety and phosphate fertilizer dose on the blood profile of Djallonke lambs

	Zaayura		Hewale		SED	*P*_*- value*_		
Blood profile	30	90	30	90		V	P rate	V x P rate
Hb g/l	10.3	10.2	10.3	9.9	0.9	0.731	0.612	0.827
PCV %	31.0	30.6	30.8	29.5	2.6	0.720	0.624	0.826
WBC Total X10^9^ L	5.9	5.7	6.5	5.8	0.8	0.584	0.410	0.635
Albumin (g/l)	26.2	24.9	24.3	24.0	1.0	0.079	0.331	0.504
Globulin (g/l)	12.8	11.3	15.1	16.1	1.9	0.023	0.859	0.387
Glucose (mmol/l)	0.6	0.5	0.8	0.64	0.2	0.113	0.283	0.871
Total Protein (g/l)	38.9	36.2	39.4	40.2	1.9	0.124	0.469	0.209
Blood urea nitrogen (mmol/l)	10.9	10.3	9.0	12.2	1.1	0.997	0.124	0.032

Means with the same letters are not significantly different (*P* > 0.05), *Hb* Haemoglobin, *PCV* Packed cell volume, *WBC* White blood cell, *Zaayura* Zaayura-SARC 4-75, *Hewale* Hewale-IT93K-192-4, *V* Variety, *P* Phosphorus fertilizer, *SED* Standard Error of Difference, *P* probability

## Discussion

The differences observed in the nutrient composition and in vitro organic matter digestibility of the V at different P doses suggests the varieties could be selected for different dose of phosphate application. The CP concentrations reported in this study compared favorably with those reported by Anele et al. [17] for other improved dual purpose cowpea. Turk et al. [18] reported an increase in CP concentration in sainfoin with increasing P dose. In this study, the CP concentration of the V responded differently to increasing P dose. The differences might have been influenced by the difference in legume specie and variety used. Leguminous plants that undergo biological nitrogen fixation are sensitive to phosphate deficiency since it is a major source of energy for *Rhizobium* bacteria to convert atmospheric nitrogen to ammonium for plant use [19]. This has the potential to significantly affect the nitrogen concentration of the cowpea haulm. The differences in CP concentration could be due to differences in the ability of the various V to take up nitrogen from the soil, since P was not limiting in this study. The CP levels reported in this study were above the adequate levels of 110-130 g/kg required for maintenance and growth of small ruminants [20] and could therefore be used as supplemental CP source to ruminants fed low quality cereal straw.

The NDF and ADF values reported in this study were lower than what was reported by Anele et al. [17] in some other improved varieties of cowpea haulms cultivated without P application. The differences may have been due to the effect of the P fertilizer dose applied. Decreasing levels of NDF and ADF have been reported in sainfoin with increasing P dose by Turk et al. [18]. This trend did not reflect in this study as different varieties were found to respond differently to increasing P doses in relation to NDF and ADF. Stage of harvesting is noted to significantly affect the deposition of plant cell wall (NDF and ADF). The vegetative growth phase of plants is usually characterised by lower NDF and ADF fraction with higher CP concentration than the reproductive or flowering growth phase [21]. The differences in NDF and ADF in the present study is an indication that the deposition of structural carbohydrates in cowpea haulms can be influenced by V and P doses even though they were all harvested at the same growth stage.

The differences in DOM among varieties could be explained by the differences in CP, NDF and ADF. The high CP may have supplied the needed degradable protein for cellulolytic microbes to digest the available organic matter.

The variation in DMI among the V at the different P doses might be attributed to the differences in CP, NDF and ADF concentrations at the different P dose. Higher ADF has been found to reduce voluntary DMI [22].

The dressing percentage reported in this study for the lambs marginally differed from the average 42.3 kg reported for Djallonke sheep slaughtered at 20 kg live weight [23]. The results from this study suggests that, supplementing the diet of Djallonke lambs grazing natural pasture with the different cowpea haulms treated with different P doses has the potential to improve the dressing percentage. The primal cuts, internal organs and external organs were higher in lambs fed haulms fertilized with a 90 kg P dose than the 30 kg P dose. This is an indication that fermentable organic matter was well synchronized with the dietary protein degraded in the rumen and therefore supplied the required volatile fatty acids (VFA) for muscle synthesis. Even though lower CP was obtained in haulms fertilized with a P dose of 90 kg/ha compared to 30 kg/ha, the BUN concentration in animals supplemented with P dose 30 kg/ha was higher, suggesting some limitation on the ability of the rumen microbes to effectively degrade the CP in the P dose 30 kg/ha compared to the 90 kg/ha. This could further explain the differences in the carcass parameters reported between P dose 30 kg/ha and 90 kg/ha.

Blood is an important index of physiological, pathological and nutritional status in living organisms [24]. The haematological values fell within the ranges reported for West African Dwarf Sheep [25, 26]. The Hb and PCV reported in this study were slightly higher than what was reported by Anele et al. [17] who fed cowpea haulm to West African Dwarf sheep. The stable Hb values reported in this study is an indication of the protein quality of the haulms. A higher BUN (*P <* 0.05) was reported in this study for Hewale-IT93K-192-4 at higher P dose indicating a better protein degradation in the rumen of the lambs on a P dose of 90 kg /ha. Globulin concentration could give an indication of the immune response of an organism [27]. The globulin concentrations reported in this study were enough to support the immune system of the lambs. The slightly higher (*P <* 0.05) globulin in Hewale-IT93K-192-4, suggest a better microbial degradation of the CP in Hewale-IT93K-192-4 than Zaayura-SARC 4-75 even though Zaayura-SARC 4-75 had a higher CP than Hewale-IT93K-192-4.

## Conclusion

The study showed a strong Variety and phosphate fertilizer dose effects on the nutrient composition, digestible organic matter and growth performance of Djallonke sheep. When selecting and cultivating dual purpose cowpea, the dose of phosphate fertilizer application should be taken into consideration. All the varieties at the various P doses have potential to be used as supplementary feed for Djallonke rams grazing natural pasture. Varieties Zaayura-SARC 4-75 and Hewale-IT93K-192-4 at a P dose of 90 kg/ha are recommended for WAD sheep supplementation to enhance slaughter and dressed weights.

### Statement of ethics approval

The component of the study that had to do with the use of animals were conducted in accordance with the ethics policy (2010) of the Institute of Continuing Education and Interdisciplinary Research (ICEIR) of the University for Development studies, Tamale, Ghana.
